# Neurocognitive and Memory-Enhancing Effect of *Tanacetum vulgare* Essential Oil: Involvement of Hippocampal Neurotrophic Signaling

**DOI:** 10.3390/pharmaceutics18040449

**Published:** 2026-04-06

**Authors:** Borislava Lechkova, Michaela Shishmanova-Doseva, Niko Benbassat, Pepa Atanassova, Nadya Penkova, Petar Hrischev, Zhivko Peychev

**Affiliations:** 1 Department of Pharmacognosy and Pharmaceutical Chemistry, Faculty of Pharmacy, Medical University of Plovdiv, 4002 Plovdiv, Bulgaria; borislava.lechkova@mu-plovdiv.bg (B.L.); niko.benbasat@mu-plovdiv.bg (N.B.); 2 Research Institute, Medical University of Plovdiv, 4002 Plovdiv, Bulgaria; 3 Center for Competence PERIMED-2, Central District, Vasil Aprilov Blvd. 15A, 4002 Plovdiv, Bulgaria; 4 Department of Pharmacology, Toxicology and Pharmacotherapy, Faculty of Pharmacy, Medical University of Plovdiv, 4002 Plovdiv, Bulgaria; 5 Department of Anatomy, Histology and Embryology, Faculty of Medicine, Medical University of Plovdiv, 4002 Plovdiv, Bulgaria; pepa.atanasova@mu-plovdiv.bg (P.A.); nadya.penkova@mu-plovdiv.bg (N.P.); 6 Department of Physiology, Faculty of Medicine, Medical University of Plovdiv, 4002 Plovdiv, Bulgaria; petar.hrischev@mu-plovdiv.bg; 7 Department of Medical Informatics, Biostatistics and E-Learning, Faculty of Public Health, Medical University of Plovdiv, 4002 Plovdiv, Bulgaria; zhivko.peychev@mu-plovdiv.bg

**Keywords:** *Tanacetum vulgare*, cognitive functions, passive, spatial and recognition memory, anxiety, BDNF, hippocampus

## Abstract

**Background**: Scientific interest has grown in naturally derived compounds capable of supporting or enhancing cognitive performance. *Tanacetum vulgare* L. is an abundant source of secondary metabolites and has been associated with a broad range of biological activities; however, its potential influence on cognitive function remains largely unexplored. **Methods**: The present study explored the effects of *T. vulgare* essential oil (EO) on cognitive performance, hippocampal brain-derived neurotrophic factor (BDNF) expression, and histomorphological alterations in a rat model. Animals were administered *T. vulgare* EO at doses of 0.5 and 1.5 mL/kg for 28 days and were subjected to a series of behavioral tests after one week of pretreatment. **Results**: Both doses of EO facilitated the formation of short- and long-term memory traces in the inhibitory avoidance tasks, with a more pronounced effect observed at the lower dose, whereas improvement in passive learning was evident only at the higher dose. Spatial and recognition memory were enhanced at both doses. EO treatment significantly increased hippocampal BDNF expression without inducing pathological alterations. **Conclusions**: These findings suggest that *T. vulgare* EO may improve specific hippocampal-dependent cognitive functions, with upregulation of hippocampal BDNF representing a potential underlying mechanism.

## 1. Introduction

Cognitive health has become an increasingly important focus within biomedical research, particularly as demographic trends show a rise in aging populations and a corresponding increase in age-related cognitive decline and neurodegenerative disorders (such as Alzheimer’s disease, Parkinson’s disease, Multiple sclerosis, and others) [1,2]. Moreover, recent studies suggest that cognitive functioning in younger populations may also be negatively affected as a result of excessive use of digital technologies, poor sleep quality, substance use, and other lifestyle and environmental stressors [3,4,5,6,7,8,9]. Cognitive decline, encompassing impairments in memory, attention, executive function, and learning capacity, poses significant challenges for public health and individual well-being [1,2,10,11,12]. Although pharmacological treatments are available, their efficacy is often limited, and many are associated with adverse effects. Consequently, there is growing scientific interest in identifying safe, naturally derived compounds that may support or enhance cognitive performance [13,14,15,16,17,18,19].

Essential oils represent a class of plant-derived products that has gained considerable scientific attention in recent years. Essential oils are complex mixtures of volatile lipophilic substances of both terpenoid and non-terpenoid origin, obtained from plant material by water or steam distillation, or by a mechanical process. The composition of many essential oils is dominated by monoterpenes, sesquiterpenes, and phenylpropanoids, and may also include nitrogen- or sulfur-containing compounds, diterpenes, and other minor constituents [20,21].

Ancient civilizations have long employed essential oils for healing and religious purposes [22]. The contemporary use of essential oils spans multiple fields, including the food industry, cosmetic and perfumery formulations, healthcare and medical applications, as well as agricultural practices [23,24,25,26,27]. In addition to their characteristic aroma, essential oils exhibit a broad spectrum of biological activities—antioxidant, antimicrobial, antiviral, anti-inflammatory, and dermatoprotective [28,29,30,31,32,33]. Recent studies indicate their efficacy in managing skin disorders (e.g., acne [34]), antitumor potential [35,36], immune-enhancing effects [37], facilitation of wound healing [38], improvement in gastrointestinal tract conditions [39,40,41], and hepatoprotective effects [42]. Moreover, essential oils have been linked to various actions on the central nervous system, exhibiting the potential to alleviate anxiety [43,44,45,46], depression [47,48], epilepsy [49,50], and dementia [51,52].

Various medicinal plants contain bioactive constituents such as flavonoids, terpenoids, and alkaloids, which have been reported to exert neuroprotective, antioxidant, and anti-inflammatory properties—mechanisms closely linked to cognitive resilience [16]. However, many botanical species and their essential oils remain insufficiently studied, and experimental evidence is needed to validate their potential effects on cognitive processes.

One such species is *Tanacetum vulgare* (*T. vulgare*), or common tansy—a perennial aromatic plant, belonging to the family Asteraceae, widely distributed across Europe, North and South America, North Africa, and Asia. Traditionally, it has been employed for a variety of medicinal purposes, including as an antihypertensive, antispasmodic, anthelmintic, and acaricidal agent. It has also been used externally for wound healing and against dandruff [53]. The species is an abundant producer of phenolics and essential oil with diverse composition, mainly including oxygenated monoterpenes [54,55].

**Figure 1:**
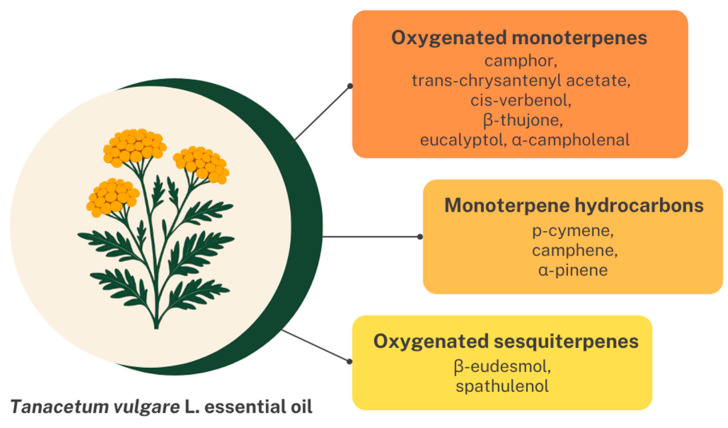
Main constituents of *Tanacetum vulgare* L. essential oil.

The chemical composition of the *T. vulgare* EO used in our study has been previously analyzed by gas chromatography–mass spectrometry (GC-MS) [56]. Figure 1 illustrates the main constituents of the EO.

The EO was predominantly composed of oxygenated monoterpenes, with camphor, trans-chrysantenyl acetate, and *cis*-verbenol identified as the major constituents in concentrations of 25.24%, 18.35%, and 10.58%, respectively. Other notable components within this group included β-thujone, eucalyptol, and α-campholenal (concentrations of 6.06%, 5.99%, and 5.98%, respectively). Regarding monoterpene hydrocarbons, *p*-cymene was present in the highest proportion (3.16%), followed by camphene and α-pinene (1.84% and 0.52%, respectively). Oxygenated sesquiterpenes were the least abundant, with *β*-eudesmol and spathulenol representing the main compounds of this fraction (0.47% and 0.36%, respectively) [56].

Although *T. vulgare* is associated with a broad spectrum of biological activities—such as antioxidant, anti-inflammatory, antimicrobial, enzyme-inhibitory, antiproliferative, and hepatoprotective effects—reports addressing its impact on cognitive function remain limited [57,58,59,60,61,62,63]. This issue is especially relevant given the growing interest in natural compounds as potential cognitive-enhancers. In addition, the mechanisms by which they may affect cognitive function are still insufficiently characterized. To address these limitations, the present study provides novel evidence on the effects of essential oil isolated from *T. vulgare* on cognitive performance and hippocampal brain-derived neurotrophic factor (BDNF) expression, as well as on potential histopathological alterations in different organs of treated animals.

## 2. Materials and Methods

### 2.1. Plant Material and Isolation of Essential Oil

Inflorescences of wild-grown *Tanacetum vulgare* L. in the western Rhodope Mountains of Bulgaria were collected and dried in a well-ventilated and shaded area at room temperature for approximately one week. The plant material was subjected to hydrodistillation for 4 h using a Clevenger-type apparatus (SIMAX, Kavalierglass, Sázava, Czech Republic) to isolate the essential oil (1.2% *w*/*w* yield).

### 2.2. Animals

In the present study, thirty adult male Wistar rats weighing 140–160 g were used. The animals were supplied by the Animal Center of the Medical University, Plovdiv and housed in plastic cages. Food and water were provided *ad libitum*. All rats were kept under standard laboratory conditions (12/12 h light–dark cycle, temperature of 21–25 °C, and humidity of 55 ± 5%). All procedures complied with the requirements of the Bulgarian Food Safety Agency (permission 456/2025) and were formally approved by the Ethics Committee on Human and Animal Experimentation of the Medical University of Plovdiv. This study was conducted in strict accordance with the guidelines of the European Community Council directive 86/609/EEC.

The rats were randomly divided into 3 groups as follows:

- Group 1 (C-veh)—treated with *Oleum Helianthi* (sunflower oil) 1 mL *p. os*;
- Group 2—treated with *T. vulgare* EO 0.5 mL/kg *p. os*;
- Group 3—treated with *T. vulgare* EO 1.5 mL/kg *p. os.*

During the entire testing period, all animals were treated daily. Each test was preceded by the administration of the EO and the vehicle, with a 30 min interval.

### 2.3. Behavioral Test

**Figure 2:**
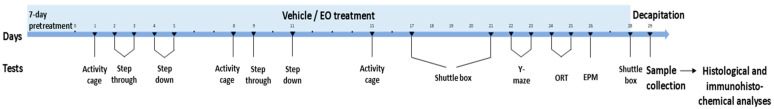
Timeline of the experiment.

Figure 2 presents the timeline of all tests performed.

#### 2.3.1. Activity Cage Test

The activity cage test was performed in a transparent plastic box (40 × 40 cm with 40 cm high walls) as previously described in detail [64]. In brief, this test was applied to assess the spontaneous locomotor activity of rats, as determined by the number of their horizontal and vertical movements, on days 1, 8, and 15 of the experiment.

#### 2.3.2. Passive Avoidance Test with Negative Reinforcement Step-Through

The step-through passive avoidance test was conducted as depicted in our previous study [65]. The device consists of two equal-sized light and dark compartments separated by an automated sliding door. The test was performed to assess the passive learning on day 1, short-term memory traces on day 2 and long-term memory traces on day 8. Latency of reactions (time spent in the light compartment for more than 178 s), was used as the criterion for learning and memory retention [66].

#### 2.3.3. Passive Avoidance Test with Negative Reinforcement Step-Down

The step-down passive avoidance test was carried out as previously described [67]. Briefly, a one-compartment device with a plastic platform above the floor grid was used. The test was conducted to investigate the passive learning on day 1, short-term memory retention on day 2, and long-term memory traces on day 8. The criterion for learning and memory retention was latency time (remaining of the animal on the platform for more than 60 s).

#### 2.3.4. Active Avoidance Test Shuttle Box

The active avoidance test was conducted in a shuttle box consisting of two chambers separated by an open gate, as previously described [67,68]. In brief, this test was conducted to assess active learning over five consecutive days, with day 12 reserved for testing memory retention. The following behavioral parameters were noted: number of avoidances, number of escapes, and number of intertrial crossings.

#### 2.3.5. Y-Maze Test

Hippocampal-dependent spatial working memory was determined using a Y-maze test according to the protocol described in our previous report [65,68]. In summary, the test was conducted in a black Perspex cage containing three arms, and the number of alternations was recorded. An alternation was defined as entering three different arms consecutively. The percentage of spontaneous alternations was calculated using the following formula: SA (%) = (number of alternations)/(total number of entries − 2) × 100.

#### 2.3.6. Object Recognition Test

The object recognition test, which consisted of an exploration session and a test session, was conducted over two consecutive days [64,65]. It was performed in an open Plexiglas box using plastic objects, as previously described [64,69]. Briefly, the test was performed to evaluate the recognition memory during the test session on day 2, when rats were allowed to explore two different objects for 5 min. The time spent exploring each object was recorded. The discrimination index (DI) was calculated as the difference in exploration time between a novel object and a familiar object divided by the total exploration time for both objects in the test session: (TB − TA)/(TB + TA).

#### 2.3.7. Elevated Plus Maze Test

This test evaluates anxiety-like behavior in rodents. The apparatus is a “+”-shaped maze elevated above the floor. It has two enclosed arms and two open arms positioned opposite each other, as well as a central area, as previously described [65]. During the test, the rodents freely explore the maze. The following parameters are recorded: the total number of entries into the arms, the number of entries into the open arms, the number of entries into the enclosed arms, and the time spent in the open arms. A preference for the open arms indicates reduced anxiety levels [64].

### 2.4. Immunohistochemistry

The day after the final behavioral test, the rats from all the experimental groups were decapitated, and the brain samples from each rat were collected for immunohistochemical analysis. The ABC method with a rabbit ABC staining system was used for the immunohistochemical reaction (Santa Cruz Biotechnology, Dallas, TX, USA) and primary antibody—anti-BDNF antibody (mouse brain-derived neurotrophic factor monoclonal antibody, MBS21105750, MyBioSurce, San Diego, CA, USA) as described in our previous report [68]. A DAB chromogen was used for visualization. The deparaffinized and Vecta mount sections were examined and photographed using a Leica DM 3000 microsystem (Leica, Wetzlar, Germany).

### 2.5. Histological Evaluation

Following euthanasia, tissue samples for histological analysis were collected from the brain and internal organs of rats from the control group and the groups treated with *T. vulgare* L. EO. Liver, kidney, heart, lung, and brain from both control and treated animals were fixed in 10% neutral formalin and embedded in paraffin as reported in our previous investigation [68]. Standard hematoxylin–eosin (HE) staining of the sections was performed (Hematoxylin H9627 and Eosin B 861006, Sigma-Aldrich, St. Louis, MO, USA). The acquired samples were examined using an Olympus light microscope (Olympus, Tokyo, Japan) and microphotographic images were obtained with a microscope camera.

### 2.6. Statistical Analysis

A statistical analysis of the results was performed using IBM SPSS^®^ (version 19.0). Data are presented as mean and the standard error of the mean (±SEM). Parametric tests were used to analyze the results because of normally distributed data, as assessed by the Kolmogorov–Smirnov test. A one-way ANOVA test was performed to analyze results from all tests. Tukey or Games–Howell *post hoc* tests were applied for intergroup differences depending on the homogeneity of variance. Differences with *p* < 0.05 were considered statistically significant.

## 3. Results

### 3.1. Activity Cage

**Figure 3:**
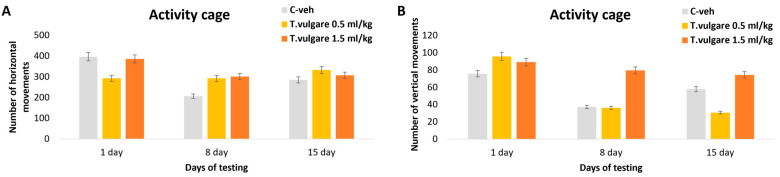
Effect of *T. vulgare* essential oil on (**A**) the number of horizontal movements and (**B**) the number of vertical movements in the activity cage.

Examination of locomotor activity revealed no significant differences between the groups treated with different doses of *T. vulgare* EO and control animals in either horizontal or vertical movements across three testing sessions conducted on days 1, 8, and 15 of the experiment (Figure 3).

### 3.2. Latency of Reaction–Learning and Memory Sessions in the Step-Through Test

**Figure 4:**
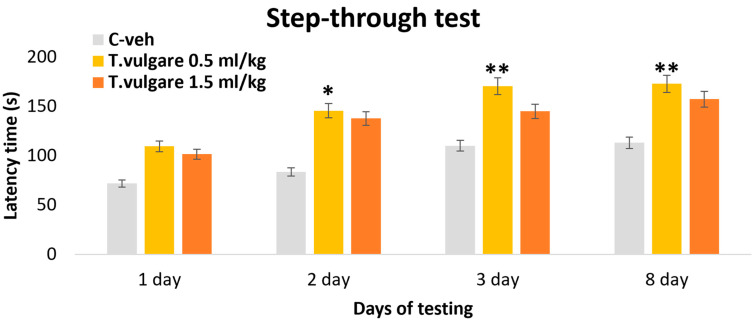
Effect of *T. vulgare* essential oil on the latency time during learning and memory in the passive avoidance step-through test. * *p* < 0.05, ** *p* < 0.01—compared to the C-veh group.

When analyzing step-through latency in the passive avoidance learning test, no statistically significant differences were observed between the experimental groups and control animals during the learning session on day 1 [F(2,29) = 2.050, *p* = 148]. ANOVA showed an effect of the treatment during day 2 [F(2,29) = 4.359, *p* = 0.023], day 3 [F(2,29) = 6.783, *p* = 0.004], and day 8 [F(2,29) = 5.823, *p* = 0.008]. On day 2, the *post hoc* test revealed that the group treated with 0.5 mL/kg *T. vulgare* EO exhibited a significantly longer time for staying in the light chamber of the apparatus, compared with the control group (*p* = 0.03) (Figure 4). During both short-term and long-term memory retention tests, animals treated with the lower dose of 0.5 mL/kg of the EO again showed significantly longer latencies than control animals (*p* = 0.003 and *p* = 0.008, respectively) (Figure 4). The group treated with 1.5 mL/kg *T. vulgare* EO demonstrated only a trend toward increased latency relative to the control group during the long-term memory retention retest (*p* = 0.055).

### 3.3. Latency of Reaction–Learning and Memory Sessions in the Step-Down Test

**Figure 5:**
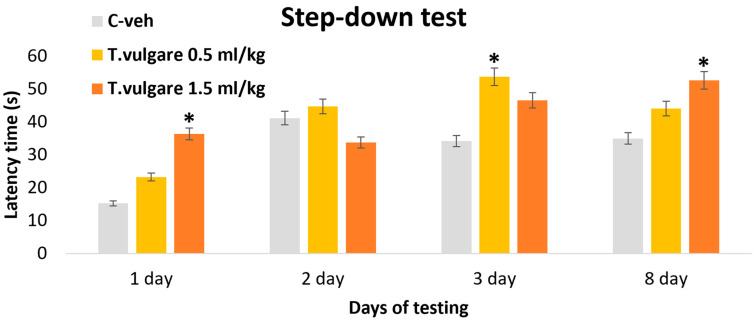
Effect of *T. vulgare* essential oil on the latency time during learning and memory in the passive avoidance step-down test. * *p* < 0.05—compared to the C-veh group.

In the passive avoidance step-down test, analysis of variance showed a significant effect during day 1 [F(2,29) = 3.468, *p* = 0.046], day 3 [F(2,29) = 3.975, *p* = 0.031], and day 8 [F(2,29) = 3.150, *p* = 0.05]. The *post hoc* test, applied for the between-group differences, revealed a significant increase in the latency of reaction time observed on day 1 of the learning session in the group treated with *T. vulgare* EO at a dose of 1.5 mL/kg, compared with control animals (*p* = 0.038). During the assessment of short-term memory, only the group treated with 0.5 mL/kg *T. vulgare* EO exhibited a significant prolongation of latency relative to the control group (*p* = 0.025) (Figure 4). During the memory retention test conducted on day 8, the group treated with the higher dose of 1.5 mL/kg *T. vulgare* EO exhibited a significantly longer time spent on the platform compared with control rats (*p* = 0.047) (Figure 5).

### 3.4. Avoidances–Learning and Memory Sessions in a Shuttle Box

**Figure 6:**
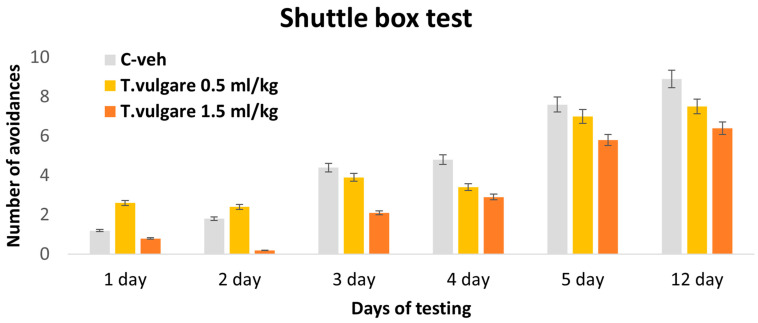
Effect of *T. vulgare* essential oil on the number of avoidances during learning and memory tests in the Shuttle box.

The results of our study showed that no statistically significant differences were observed between the control group and animals treated with different doses of *T. vulgare* EO throughout all five days of the learning session, as well as during the long-term memory retest conducted on day 12 (Figure 6).

### 3.5. Escapes–Learning and Memory Sessions in a Shuttle Box

**Figure 7:**
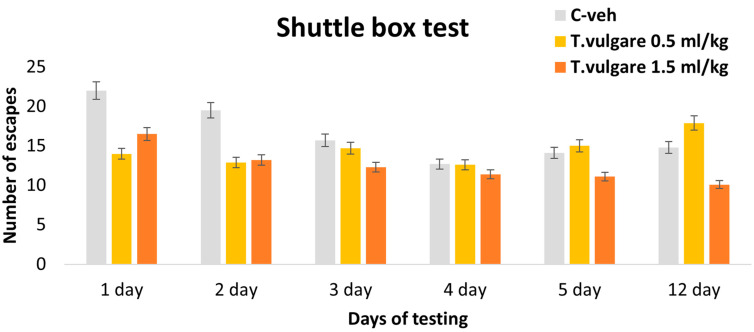
Effect of *T. vulgare* essential oil on the number of escapes during learning and memory tests in the Shuttle box.

The *post hoc* test revealed no significant differences in the number of escapes between the control group and animals treated with different doses of *T. vulgare* EO (Figure 7).

### 3.6. Intertrial Crossings–Learning and Memory Sessions in the Shuttle Box

**Figure 8:**
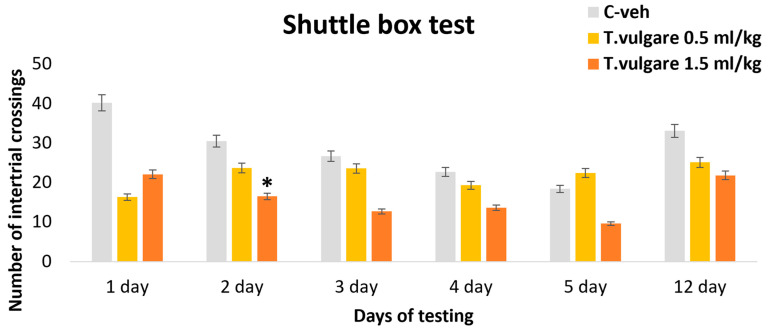
Effect of *T. vulgare* essential oil on the number of intertrial crossings during learning and memory tests in the Shuttle box. * *p* < 0.05—compared to the C-veh group.

During the active avoidance test with negative reinforcement, we found that the number of intertrial crossings in the experimental group treated with 1.5 mL/kg *T. vulgare* EO was significantly lower than that in the control group only on day 2 of the learning session (*p* = 0.022) (Figure 8). No significant differences in the number of intertrial crossings between the groups were observed during the memory retention test.

### 3.7. Spatial Memory Assessed in the Y-Maze Test

**Figure 9:**
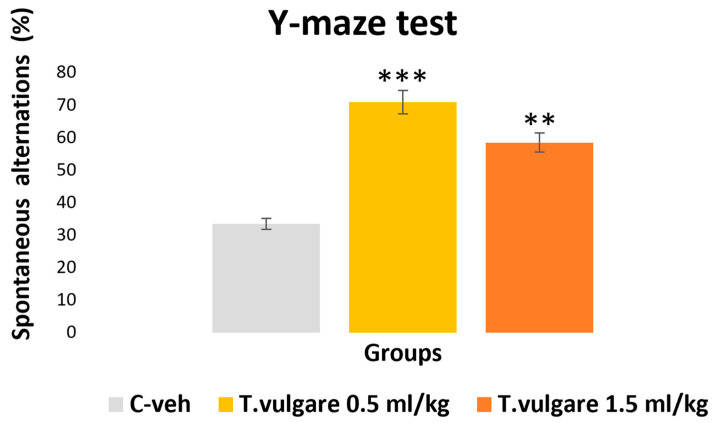
Effect of *T. vulgare* essential oil on spontaneous alternations in the Y-maze test. ** *p* < 0.01, *** *p* < 0.001—compared to the C-veh group.

ANOVA demonstrated a significant treatment effect [F(2,29) = 12.634, *p* < 0.001] in the Y-maze test. The *post hoc* test revealed that both groups treated with different doses of *T. vulgare* essential oil had a significantly higher percentage of spontaneous alternations than the control group: 0.5 mL/kg *T. vulgare* vs. C-veh group, *p* < 0.001; 1.5 mL/kg *T. vulgare* vs. C-veh group, *p* = 0.008 (Figure 9).

### 3.8. Recognition Memory Assessed in the Object Recognition Test

**Figure 10:**
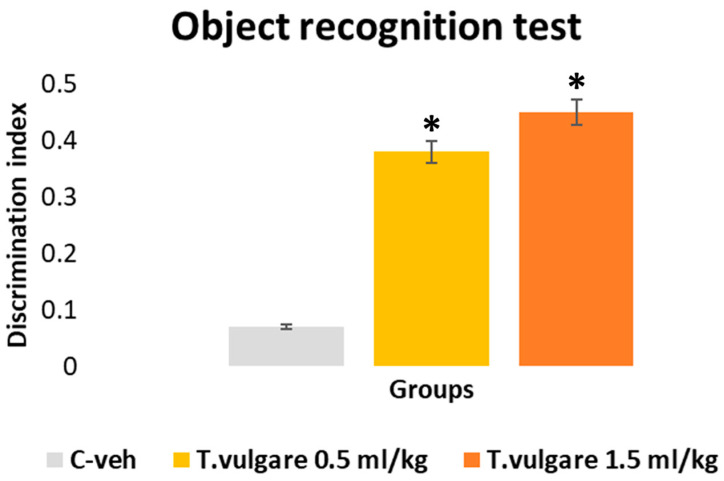
Effect of *T. vulgare* essential oil on recognition memory. * *p* < 0.05—compared to the C-veh group.

Analysis of variance revealed a treatment effect [F(2,29) = 5.698, *p* = 0.008]. A statistically significant difference in the discrimination index was revealed between the three experimental groups. Animals treated with different doses of *T. vulgare* EO (0.5 mL/kg and 1.5 mL/kg) demonstrated enhanced recognition memory compared with the control group (*p* = 0.024 and *p* = 0.013, respectively) (Figure 10).

### 3.9. Anxiety Index Assessed in the EPM Test

**Figure 11:**
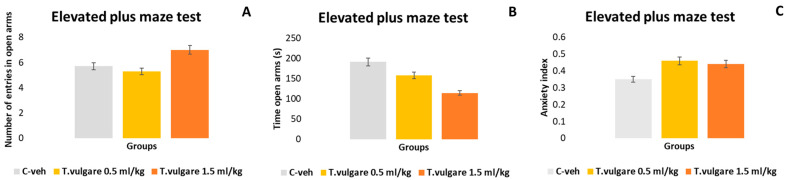
Effect of *T. vulgare* essential oil on (**A**) number of entries in the open arms of the EPM test; (**B**) time spent in the open arms of the EPM test; (**C**) anxiety index.

Analysis of the anxiety index (AI) in the elevated plus maze (EPM) revealed no significant differences among the three experimental groups in either the number of entries into the open arms or the time spent in them (Figure 11A–C). No significant differences were observed for the overall anxiety index.

### 3.10. Histological Results

**Figure 12:**
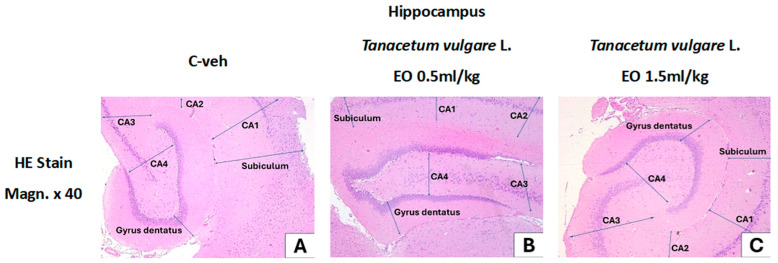
Hippocampal formation of rats from the control and treated groups: dental gyrus, hippocampus (CA1, CA2, CA3, CA4 regions), and subiculum, stained with HE. Hippocampus with normal structure. The hippocampus is a major part of the hippocampal formation. It is a deep cortical fold formed by the incising of the sulcus hippocampi, which projects along the inferior medial wall of the inferior horn of the lateral ventricle. The hippocampus is covered by a plate of white matter, the alveus, which isolates it from the cavity of the lateral ventricle. The dentate gyrus is a narrow, serrated cortical fold parallel to the fimbria hippocampi, located between it and the sulcus hippocampi. Anteriorly, the dentate gyrus passes along the free surface of the uncus of the parahippocampal gyrus. The subiculum is a direct continuation of the hippocampus to the cortex of the gyrus parahippocampalis and its entorhinal area. (**A**) Hippocampus—controls, (**B**) hippocampus—*T. vulgare* L. EO 0.5 mL/kg, (**C**) hippocampus—*T. vulgare* L. EO 1.5 mL/kg.

Figure 12 presents a routine histological examination with HE staining of the hippocampus in rats treated with *T. vulgare* EO.

The images of the histological sections show normal structure of the hippocampus in control rats and in rats treated with different concentrations of *T. vulgare* EO, divided into two groups: first group—*T. vulgare* EO 0.5 mL/kg, and second group—*T. vulgare* EO 1.5 mL/kg.

The hippocampal slices of animals from the three groups demonstrated a normal structure of the hippocampal formation with its parts: hippocampus, dentate gyrus, subiculum, and parasubiculum. No abnormalities were observed in the five-layered cortex of the hippocampus, which consists of three cellular and two fibrous layers (Figure 12A–C).

**Figure 13:**
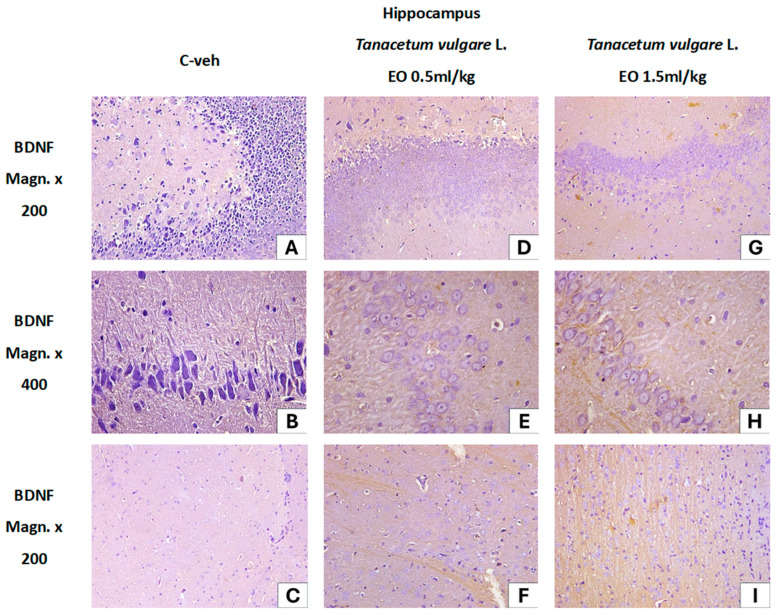
The immunohistochemical reaction for BDNF in hippocampus of rats from the control and treated groups. Negative immunohistochemical reaction for BDNF in the hippocampus of the control group in neurons and nerve fibers from C4 region and dental gyrus (**A**–**C**). Positive immunohistochemical reaction for BDNF in the hippocampus of rats from both experimental groups: *T. vulgare* L. EO 0.5 mL/kg. BDNF-positive neurons with moderate intensity of expression, evenly distributed in the cell layers of the dentate gyrus. BDNF-positive nerve fibers form loose, parallel bundles (**D**–**F**). *T. vulgare* L. EO 1.5 mL/kg. Groups of BDNF-positive neurons with strong immunohistochemical reaction in the white matter of the CA4 region. Strong reaction in neurons from the cell layers of the dentate gyrus. Dense BDNF-positive neuronal network (**G**–**I**).

The immunohistochemical analysis for BDNF in the hippocampal sections is presented in Figure 13. In the control group, the BDNF reaction was negative. No BDNF expression was observed in the neuronal and fibrous layers of the hippocampus, dentate gyrus, or subiculum (Figure 13A–C).

The immunohistochemical reaction for BDNF in the hippocampal sections of rats from the experimental groups treated with the EO was positive. Endogenous BDNF was expressed in various cellular components—in the perikaryons, intranuclearly, in the nerve fibers of the cortical layers, as well as in the white matter of the hippocampal regions CA1, CA2, CA3, and CA4.

In the hippocampus of the first group of experimental rats treated with *T. vulgare* L. EO (0.5 mL/kg), BDNF-positive neurons with moderate reaction intensity were observed. They were evenly distributed across the layers of the dentate gyrus—superficial, molecular layer; middle, granule cell layer; and deep, polymorphic layer. Loose bundles of parallel BDNF-positive nerve fibers were also observed in the white matter of the CA4 region (Figure 13D–F).

In the hippocampus of the second group of experimental rats treated with *T. vulgare* L. EO (1.5 mL/kg), the intensity of the reaction was significantly higher than in the animals from the first group. Strongly positive BDNF-active neurons with intense cytoplasmic staining of the perikaryon were unevenly distributed across different regions of the hippocampal formation. In the dentate gyrus, among the large number of BDNF-positive neurons, a dense BDNF-positive neuronal network with bundles of nerve fibers in different directions was established. Intense staining of cell bodies was also noted within the BDNF-positive neuronal network of the CA4 region (Figure 13G–I).

**Figure 14:**
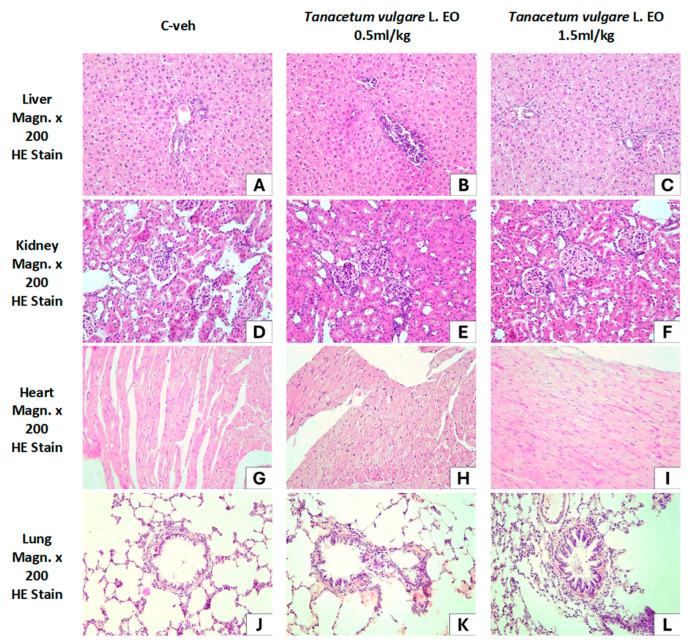
Internal organs, HE-stain, Magn.×200. (**A**) Liver C-veh, controls; (**B**) liver *T. vulgare* EO 0.5 mL/kg; (**C**) liver *T. vulgare* EO 1.5 mL/kg. (**D**) Kidney C-veh, controls; (**E**) kidney *T. vulgare* EO 0.5 mL/kg; (**F**) kidney *T. vulgare* EO 1.5 mL/kg. (**G**) Heart C-veh, controls; (**H**) heart *T. vulgare* EO 0.5 mL/kg; (**I**) heart *T. vulgare* EO 1.5 mL/kg. (**J**) Lung C-veh, controls; (**K**) lung *T. vulgare* EO 0.5 mL/kg; (**L**) lung *T. vulgare* EO 1.5 mL/kg.

Figure 14 presents the morphological characteristics of internal organs: liver, kidney, heart, and lung. The histological picture of the microscopic slides with routine HE staining in all three groups of experimental animals, Group 1 (C-veh, controls), Group 2 (*T. vulgare* EO 0.5 mL/kg), and Group 3 (*T. vulgare* EO 1.5 mL/kg), shows a normal structure, without pathological changes.

Figure 14A–C show normal liver structure of the studied rats. Liver lobes with approximately hexagonal shape and radial plates of hepatocytes are observed, in relation to v. centralis and portal spaces with transverse or longitudinal sections of elements of the hepatic triad—interlobular artery, vein, and bile duct. No inflammatory infiltrates, steatosis in hepatocytes, or fibrosis are detected.

Figure 14D–F visualize the renal parenchyma. In all three groups of experimental animals, no pathological changes are observed in either the cortex or the medulla of the kidney. In the Malpighian corpuscles, the squamous epithelium of the Bowman’s capsule and the capsular space, the glomeruli of fenestrated capillaries, with erythrocytes in them and adjacent mesangial cells, are visible. The curved parts of the proximal tubules have epithelial cells with well-stained cytoplasm, and the distal tubules are made up of cuboidal epithelium with a clearly visible lumen.

Figure 14G–I demonstrate the myocardium of the heart wall without pathological changes in the three groups of rats. Cardiomyocytes form bundles of different orientation with capillaries between them. In longitudinal sections, elongated cardiomyocytes are observed, connected in a functional syncytium. No hypertrophic cardiomyocytes, inflammatory infiltration, or adipocytes are observed.

Figure 14J–L show the microscopic structure of the lung of the studied rats without pathological changes in all three groups. A well-preserved lung parenchyma with a respiratory part, bronchi, and blood vessels is observed. In the terminal bronchioles, the single-row cubic ciliated epithelium forms folds, under which a thin layer of smooth muscle cells is visible, is obeserved.

## 4. Discussion

The current study investigated the effects of *T. vulgare* EO on various aspects of behavior and cognitive functions using well-established and validated tests. No inhibitory effects on locomotor activity were observed, as determined by the rats’ vertical and horizontal movements. Hippocampal-dependent learning and memory performance were assessed using two passive avoidance tests (step-through and step-down) and a spatial Y-maze test [70]. Both doses of *T. vulgare* EO facilitated the formation of short- and long-term memory traces in the inhibitory avoidance tasks, with a more pronounced effect observed at the lower dose. However, only the higher dose of the EO exerted a positive impact on passive learning. The Y-maze test, which is based on rodents’ natural exploratory behavior, was performed to assess the spatial working memory [71]. Both doses of the EO improved performance in the spatial memory task by increasing the number of spontaneous alternations. Although the role of the hippocampus in spatial learning and memory is well known, recent studies have demonstrated that its dorsal subregions are specifically involved in spatial acquisition and consolidation [72]. Recognition memory, another cognitive domain involving the hippocampus, was assessed using the object recognition test (ORT). This task is based on rodents’ spontaneous preference for novelty, whereby novel objects are explored more than familiar ones [73]. Both doses of *T. vulgare* EO enhanced the discrimination index, which is associated with an improvement in recognition memory. In contrast, the EO had no effect on active learning and memory, as evaluated by the two-way active avoidance test performed in the Shuttle box where multiple brain structures such as the hippocampus, amygdala, and frontal cortex play a pivotal role. Moreover, no anxiolytic-like behavior was observed following *T. vulgare* EO administration in the EPM test. Taken together, these results suggest that *T. vulgare* EO may improve certain hippocampal-dependent cognitive functions.

Essential oils rich in monoterpenes, as well as isolated monoterpenes, have been extensively investigated for their neurobehavioral properties. A number of studies indicate that these compounds exert anti-inflammatory and neuroprotective effects, enhance learning and memory processes, and display anxiolytic activities [74,75,76,77,78]. These findings suggest that such compounds may modulate key mechanisms underlying cognitive function, highlighting their potential relevance for age-associated cognitive decline and neurodegenerative disorders, such as Alzheimer’s and Parkinson’s diseases.

Our previous GC-MS investigation of the EO from *T. vulgare* revealed monoterpenes as the predominant class of compounds and identified camphor (25.24%), *trans*-chrysantenyl acetate (18.35%), and *cis*-verbenol (10.58%), as the major constituents [56], which may contribute to the neurobiological effects observed in the current study.

Previous studies provide evidence that camphor, the predominant component of the tansy EO, exerts neuroprotective and neurobehavioral effects through modulation of oxidative stress, neuroinflammation, and synaptic signaling pathways. In a model of cadmium-induced neurotoxicity, Hamdollahi et al. demonstrated that camphor significantly ameliorated spatial memory deficits, an effect linked to hippocampal protection and enhanced BDNF expression. The cognitive improvement was accompanied by an attenuation of oxidative stress and inflammatory responses, including restoration of glutathione levels and enhancement of antioxidant enzyme activities. Furthermore, camphor increased alpha-7 nicotinic acetylcholine receptor expression, decreased acetylcholinesterase activity, and reduced neuronal loss in the CA1 region [79].

Findings by Salama et al. further support the neuroprotective potential of camphor, demonstrating its antidepressant-like effects in a ciprofloxacin-induced depression model in rats. Camphor increased the antioxidant capacity, while reducing the levels of TLR4, TNF-α, nitric oxide, and malondialdehyde. In addition to its antioxidant and anti-inflammatory actions, camphor upregulated the P190-RHO GTP protein, which was associated with improvements in locomotor activity and histopathological changes in brain tissue [80].

Regarding *T. vulgare*, investigations for its effects on cognitive functions are limited. Daneshmand et al. demonstrated improved spatial learning and memory in a rat model of sporadic Alzheimer’s disease following treatment with *T. vulgare*, *Urtica dioica,* and *Rosa canina*, as assessed by the Morris water maze [81]. In line with these findings, Vafaee et al. reported that administration of the aforementioned extract combination protected cortical neurons and significantly improved motor performance in rats subjected to cerebral ischemia [82].

In the current study, we examined the effects of *T. vulgare* EO on hippocampal BDNF expression as a potential mechanism underlying the observed cognitive-enhancing effects. Increased endogenous BDNF expression was detected in various cellular components including the perikaryons and nerve fibers of the cortical layers, as well as the white matter of the CA1, CA2, CA3, and CA4 hippocampal regions. The lower dose of *T. vulgare* EO induced a moderate reaction intensity in BDNF-positive neurons, whereas the higher dose elicited a significantly stronger reaction. Strongly positive BDNF-active neurons were unevenly distributed across different regions of the hippocampal formation. In the dentate gyrus, a dense BDNF-positive neuronal network with bundles of nerve fibers in different directions was observed.

BDNF belongs to the neurotrophins family and is the neurotrophin with the highest expression in various brain structures. BDNF acts by binding to and activating the TrkB receptor (Tropomyosin receptor kinase B), a crucial mechanism for neuronal survival, growth, and plasticity. In the mammalian brain, the highest neuroanatomical expression of BDNF and its TrkB receptor is found in the hippocampus, particularly in the pyramidal cells of the CA1 and CA3 hippocampal fields, as well as in the granule cells of the dentate gyrus [83]. BDNF has been shown to play a crucial role in spatial learning and memory. The neurotrophic factor is a key regulator of acquisition, consolidation, and the subsequent recall of information. The modulation of synaptic transmission and plasticity by BDNF occurs through its action in different spatial and temporal domains [84]. Furthermore, BDNF is essential for regulating neuronal development and differentiation in both the young and adult brain and plays a crucial role in the process of long-term potentiation (LTP) in the hippocampus, thereby regulating cellular processes that underlie complex behaviors and cognitive functions [85]. Hippocampal neuronal circuits process multisensory information streams that include olfactory, visual, and auditory data. This sensory information is encoded as long-term memories that are crucial for adaptive behavior in response to environmental changes [86,87]. Through the process of LTP, short-term experiences are consolidated into long-term memory. LTP in the hippocampus is a cellular mechanism of learning and memory through the persistent strengthening of synapses with recent activity [88]. Long-term depression (LTD) is another key element of synaptic plasticity in the hippocampus. LTP, together with LTD, balances synaptic efficiency, allowing memory updating, forgetting, and preventing synaptic strength saturation [89].

In addition to its involvement in synaptic plasticity and LTP in the adult brain, BDNF stimulates neurogenesis in the dentate gyrus. The continuous generation of new neurons throughout life is pivotal for learning and memory formation and dealing with stress [90,91]. Along these lines, the present findings suggest that the observed improvements in hippocampal-dependent cognitive skills were accompanied by molecular changes involving increased hippocampal BDNF expression. In our previous study, tansy alcohol extract was also found to have a positive influence on learning and memory, to enhance spatial memory performance, and to increase hippocampal BDNF expression [68]. Clinical and experimental studies highlight the importance of BDNF expression in cognitive and memory impairments, suggesting BDNF as a potential target for the prevention of some neurodegenerative diseases [92].

A noteworthy observation from our study is that the neurocognitive improvements were more evident at the lower dose of *T. vulgare* EO, even though the higher dose led to a greater increase in hippocampal BDNF expression. Although oxidative processes were not assessed in this work, this pattern suggests a possible biphasic, dose-dependent action of the essential oil constituents. These findings highlight the importance of dose optimization and warrant further studies to clarify the dose–response relationship and other potential mechanisms underlying the neurocognitive effects.

Moreover, no histopathological alterations were observed in the outer morphology and histological structure of the main organs involved in xenobiotic elimination and detoxification in rats treated with the essential oil. The present findings, together with previously reported in vivo toxicity studies of the plant [56,68,93], support further research into the potential applications of tansy and its products.

## 5. Conclusions

The present findings indicate that *T. vulgare* essential oil may exert beneficial effects on specific cognitive domains, particularly passive learning, spatial recognition, and memory. Importantly, no signs of organ toxicity were observed, and the treatment with the EO showed no other evidence of adverse effects. The reported increase in hippocampal BDNF expression indicates that modulation of BDNF signaling could contribute to these effects. While the results support the potential of the essential oil for cognitive health, further studies are needed to confirm its efficacy, safety profile, and to elucidate the underlying mechanisms.

## Data Availability

The original contributions presented in this study are included in the article. Further inquiries can be directed to the corresponding author.

## Abbreviations

The following abbreviations are used in this manuscript:

EO	Essential oil
BDNF	Brain-derived neurotrophic factor
EPM	Elevated plus maze
AI	Anxiety index
ORT	Object recognition test
HE	Hematoxylin-eosin
LTP	Long-term potentiation
LTD	Long-term depression
*p. os*	*Per os*
